# Dermoscopy use in primary care: a qualitative study with general practitioners

**DOI:** 10.1186/s12875-022-01653-7

**Published:** 2022-03-15

**Authors:** Jonathan A. Fee, Finbar P. McGrady, Nigel D. Hart

**Affiliations:** Centre for Medical Education, School of Medicine, Dentistry and Biomedical Sciences, Queen’s University Belfast (QUB), Belfast, Northern Ireland UK

**Keywords:** General Practice, Family Practice, Dermoscopy, Dermatology, Melanoma, Qualitative research

## Abstract

**Background:**

Skin assessments constitute a significant proportion of consultations with family physicians (commonly called general practitioners or GPs in the UK), and referrals to hospital dermatology departments have risen significantly in recent years. Research has shown that dermoscopy use may help GPs to assess and triage skin lesions, including suspected skin cancers, more accurately. However, dermoscopy is used by a small minority of GPs in the UK. Previous questionnaire studies have aimed to establish in a limited way some perceptions of dermoscopy among GPs: this study aimed to explore more deeply the factors influencing the use of dermoscopy among GPs.

**Methods:**

This was a qualitative interview study set in UK general practice. A purposive sample was taken of GPs who were established dermoscopy users, GPs who had recently adopted dermoscopy, and those who did not use dermoscopy. A total of twelve semi-structured interviews were conducted. Audio-recordings were transcribed verbatim and analysed using a thematic analysis (Braun and Clarke).

**Results:**

GPs’ capability to use dermoscopy necessitated receiving adequate training, while previous dermatology experience and support from colleagues were also considered factors that enabled dermoscopy use. The impact of dermoscopy on patient consultations about skin complaints was generally considered to be positive, as was having an ‘in-house’ dermoscopy user within a GP practice to refer patients to. However, training in dermoscopy was not considered a priority for many GPs either due to other more pressing concerns within their practices or the perceived complexity of dermoscopy, alongside barriers such as equipment costs. Significant ethical concerns with posting patient photographs online for training and teaching purposes were also highlighted.

**Conclusions:**

Both GPs who use dermoscopy, and those who do not, consider it to have an important role in improving skin assessments within primary care. However the need for adequate training in dermoscopy and dermatology more generally was highlighted as a key barrier to its wider use. The development of competency standards for the use of dermoscopy could allow the adequacy of training to be assessed and developed.

## Background

Skin problems are estimated to constitute up to 20% of consultations with family physicians (commonly called general practitioners or GPs) in the UK [1]. The incidences of melanoma and non-melanoma skin cancers have increased markedly across much of Europe, North America and Australia over the last few decades [2, 3], and public health campaigns have encouraged awareness of new or changing skin lesions among the public [4]. GPs play an important role in the early detection of skin cancer, particularly in healthcare systems like the UK’s where GPs act as ‘gatekeepers’ to specialists such as dermatologists, and have the potential to detect concerning skin lesions opportunistically when patients are consulting about other problems. Indeed a French study reported that almost half of melanomas diagnosed in primary care were detected by GPs without the patient consulting specifically about the concerning skin lesion [5].

The number of referrals to dermatology outpatient clinics has also risen significantly: in England the total number of referrals to dermatologists in the National Health Service (NHS) for skin complaints of all types increased by almost 29% between 2008 and 2019, to over 1 million per year [6], out of an estimated English population of 56.3 million [7]. Approximately 50% of these dermatology referrals were for suspected skin cancers [8], yet only 6.5% of these suspected cancer referrals ended with a diagnosis of skin cancer [9]. A similar issue with the referral of large numbers of benign lesions as suspected skin cancers by GPs has previously been identified in the Netherlands [10], and a more recent Belgian study found that when presented with images of skin lesions, a majority of GPs felt that some benign lesions needed referred for excision [11]. This raises the question of whether some suspected cancer referrals could be avoided or managed within a general practice setting.

**Fig. 1:**
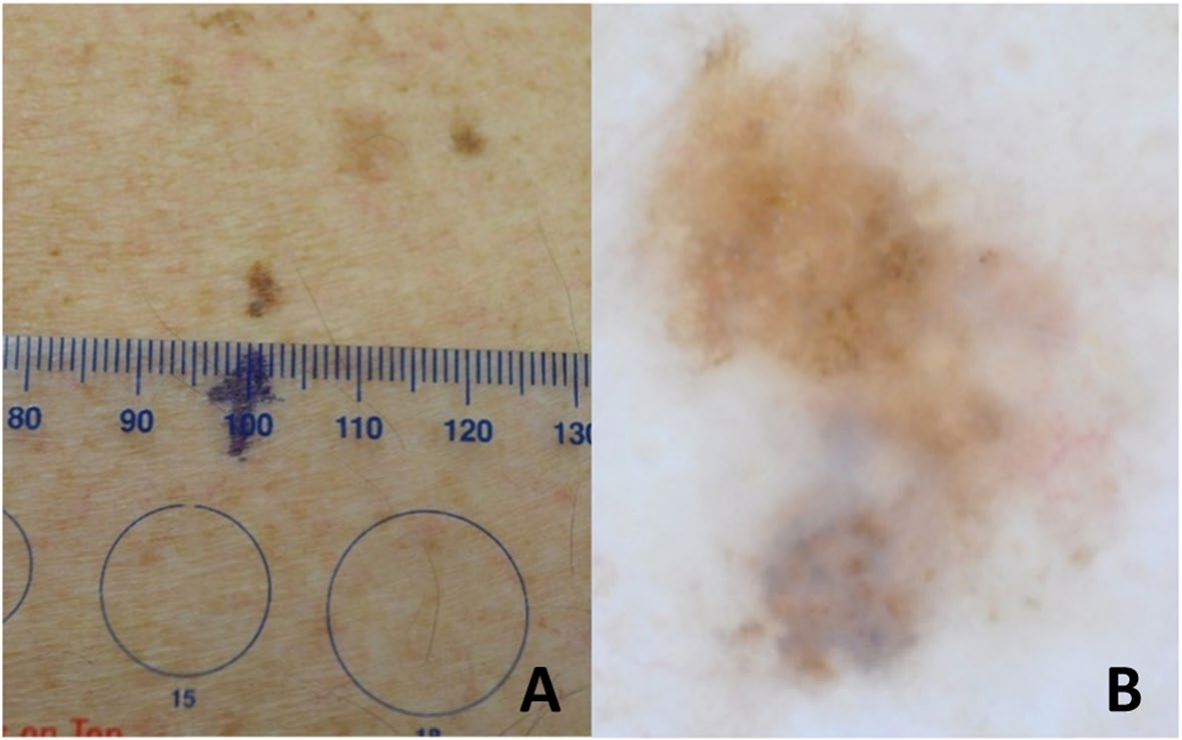
A pigmented skin lesion shown as **A** a clinical image; and **B** a dermatoscopic image. Despite the lesion’s small size, the dermatoscopic image demonstrates its asymmetry of colour and blue-grey structures in its inferior half, both of which identify it as a concerning skin lesion. The lesion was subsequently confirmed to be a melanoma. (Images courtesy of Dr Finbar McGrady)

Dermoscopy is well-established among dermatologists as an assessment tool [12], and is a non-invasive technique for the visual inspection of skin lesions. A dermatoscope is a hand-held device that contains an integral light source and typically a 10x magnification lens. A dermatoscope acts to magnify a lesion and reduce the interference of light reflection from the skin’s surface, to allow pigmented and vascular structures in the deep epidermis and superficial dermis to be visualised (see Fig. 1) [13, 14]. There is evidence from systematic reviews that skin lesion assessment using dermoscopy is more accurate than visual inspection alone for the detection of melanomas and basal cell carcinomas, especially in a secondary care setting [15, 16]. However there is also evidence that this improvement in diagnostic accuracy is only seen when it is used by doctors who are experienced in its use, highlighting the necessity of dermoscopy training [17, 18].

Evidence from the literature would suggest that the use of dermoscopy among GPs could improve their triage and assessment of skin lesions [19–21], reduce unnecessary excisions or referrals [22, 23], and that its use in primary care could be cost-effective [24]. Despite this, dermoscopy is used by a small minority of GPs in many jurisdictions including the UK, USA and France [25–28]; while reported use is higher in Australia [29]. Questionnaire studies have attempted to highlight some perceptions of GPs around the benefits of and barriers to dermoscopy use in primary care [25, 26, 30], but the methodological constraints of questionnaire studies limit the depth of understanding possible. There have been calls to make dermoscopy a standard piece of medical equipment, similar to a stethoscope or ophthalmoscope, that all clinicians have a relevant degree of experience and competence in using [31] and organisations such as the Primary Care Dermatology Society (PCDS) have recommended that at least one GP in each UK GP practice should be able to use dermoscopy [32]. Dermoscopy use is included in the GP specialty training curriculum in Australia [33], but not in other countries such as the UK [34]. If dermoscopy use in general practice is to be encouraged, understanding the factors currently influencing GPs’ use of dermoscopy is necessary.

Therefore this study asked: what factors influence GPs’ use of dermoscopy in primary care?

## Methods

A qualitative methodology was used to address the study question, and the study was underpinned by four qualitative research principles derived from grounded theory: [35] an iterative study design; purposive sampling; constant comparison of data; and data sufficiency.

The study was conducted within a post-positivist paradigm, seeking to uncover an objective truth, while acknowledging the influence of subjectivity on researchers, participants, data collection and analysis [36]. The consolidated criteria for reporting qualitative research (COREQ) were used to guide the completion and reporting of this study [37].

### Research team

The research team consisted of two GPs (NH and FM) and a GP trainee (JF). NH and FM used dermoscopy in clinical practice. FM ran occasional dermoscopy training events for GPs and worked part-time in a hospital dermatology department. NH had previous experience in conducting qualitative research and thematic analysis. JF had attended training courses on conducting and analysing qualitative interviews.

### Study setting

The study was conducted in the UK. Participant inclusion criteria were clinically active GPs practising in Northern Ireland (NI). GPs who did not work in primary care were excluded, though GPs working part-time outside primary care were eligible. GP trainees were excluded. Ethical approval was granted by Queen’s University Belfast School of Medicine, Dentistry and Biomedical Sciences Joint Research Ethics Committee in July 2018 (Ref: 18.39v2). All methods were performed in accordance with the relevant guidelines and regulations.

### Recruitment and sampling

A mixed recruitment strategy was utilized. Firstly, GPs were asked to express interest in participation by one post in August 2018 onto the newsfeeds of two online groups exclusively for NI GPs. Additional participants were recruited through snowball sampling, having been identified by other participants or GPs who knew about the study. Written informed consent was obtained from all the participants.

A purposive sample of three groups of GPs was taken defined by their use of dermoscopy: established dermoscopy users (defined as using dermoscopy for >1 year), new dermoscopy users (dermoscopy use of <1 year) and dermoscopy non-users. Variation was also sought in terms of GPs’ gender, age, years of experience working in general practice, working pattern, employment status (e.g. practice partners or temporary ‘locum tenens’ GPs), and rurality of practice (defined by NI Statistics and Research Agency’s population settlement size bands) [38]. No participant withdrew during the study. Written informed consent was obtained from all the participants.

### Data collection

Semi-structured interviews were used to collect data. An interview schedule was developed, informed by the findings of a previous scoping literature review [39] and piloted prior to use.

Interviews were conducted by JF at a location of participants’ choice. Eight interviews were carried out in participants’ places of work, three face to face in other locations, and one using teleconferencing software. Interviews were conducted between September 2018 and March 2019 and were audio-recorded. Interviews lasted between 14 and 37 min (median 19.5 min). JF kept field notes during data collection.

### Data analysis

Data analysis used a thematic analysis (Braun and Clarke) [40]. JF transcribed all interviews verbatim using Express Scribe (NCH Software Pty Ltd, Canberra, Australia). Material that identified participants or third parties was redacted prior to analysis. A copy of the transcription was sent to each participant for comment. JF and NH coded anonymized transcripts independently. JF coded transcripts using NVivo 12 Pro software (QSR International Pty Ltd, Melbourne, Australia) and NH coded by hand. After all transcripts had been coded, earlier transcripts were reviewed to determine whether codes generated from later transcripts were relevant. Both sets of codes were amalgamated with revision to remove similar codes; JF led this process with input from NH where there was uncertainty. Discussion resolved any uncertainties.

Codes were subsequently examined to generate themes. An inductive ‘bottom-up’ approach was taken to identify themes [40]. Both JF and NH were involved in this process and a thematic map was generated. A report on the qualitative analysis was sent to all participants for comment.

### Reflexivity

All participants were aware of the interviewer’s (JF’s) professional background. Some members of the research team knew some participants professionally. However as a GP trainee, JF’s prior interactions with the participants had been limited. Attempts to minimize the influence of preconceptions within the research team included regular meetings throughout the study, removing identifiable material from transcripts prior to data analysis, and involving two researchers in data analysis.

## Results

### Sample description

Twelve GPs participated in the study, whose baseline personal and practice characteristics are shown in Table 1. Three participants had experience of working in specialist dermatology posts. Most participants were partners in GP practices. Participants worked across NI in a range of urban and rural practices (for GPs working in more than one practice, the location of the majority of their work was used for defining practice variables).

**Table 1:** Description of study participants and their GP practices

**Variable**	**Number**
**Gender:**	
Male	6
Female	6
**Age (years):**	
30-39	4
40-49	4
50-59	3
60-69	1
**Length of GP experience (years):**	
<10	4
10-19	5
20+	3
**Length of dermoscopy use (years):**	
Not used	4
<1	4
1-9	1
10+	3
**GP employment status:**	
Partner	10
Other	2
**Work in general practice:**	
Full-time (7+ sessions/week)	7
Part-time (<7 sessions/week)	5
**Work in specialist dermatology post:**	
Current	2
Past	1
None	9
**Size of town where practice located:**	
80,000+ residents	2
10,000-80,000 residents	5
5,000-10,000 residents	2
<5,000 residents	3
**Total participants**	**12**

### Overview of themes

**Fig. 2:**
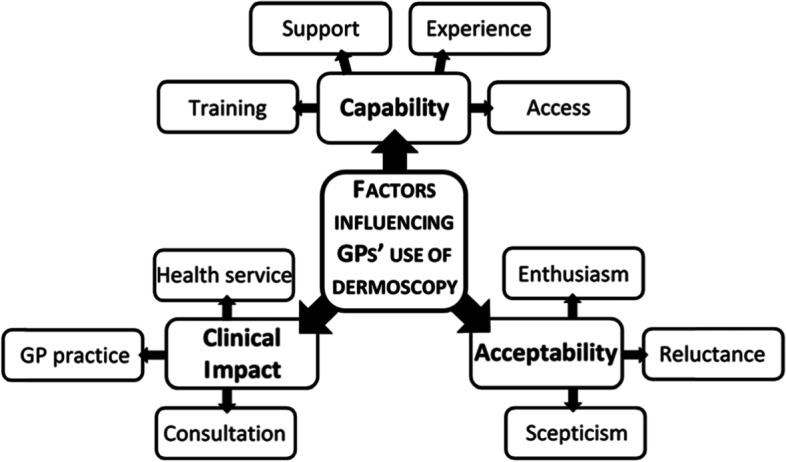
Overview of themes derived from thematic analysis

Three major themes to explain the factors influencing GPs’ use of dermoscopy were generated, each comprising several minor themes, as shown in Fig. 2.

### Capability

A key factor influencing participants’ use of dermoscopy was whether they felt capable of using it clinically. The determinants of capability included training in dermoscopy, support in practice, experience and access to dermoscopy.

#### Training

Training was considered to be essential for the effective use of dermoscopy in practice: *‘[Dermoscopy] requires…a sort of decent amount of training…before you would be able to look at these lesions properly.’(I4)*. GPs acknowledged an increase in the availability of dermoscopy training in recent years, but the accessibility of training was influenced primarily by cost: *‘Unless there was funding for [dermoscopy training] I couldn’t see myself doing that.’(I5)*. Some participants described the inadequacy of training experiences that acted as a barrier to dermoscopy use: *‘There was a day course…which was just completely baffling…I wasn’t really inspired to do much beyond that.’(I7)*.

Participants discussed the qualities that constituted adequate training in dermoscopy, which were that it was kept simple, practical, contextualised within dermatology skills more generally, and spaced out over several sessions rather than a one-off training session (Table 2).

**Table 2:** Qualities of adequate dermoscopy training derived from thematic analysis with illustrative quotations

Quality of Adequate Training		Example Quotation
Simple	*‘If it’s kept quite simple…at a GP level of knowledge, as opposed to making us dermatologists.’(I7)*	
Practical	*‘It needs to be more hands-on…bring your scopes…get people practising.’ (I6)*	
Contextualized	*‘Dermoscopy isn’t a tool on its own, it’s a tool along with…your history and your exam and all that sort of stuff’(I11)*	
Spaced	*‘Come back for another training session after a while…there has to be an on-going training thing.’(I3)*	

#### Support

Participants felt that a source of on-going support, in addition to formal training, was important in facilitating dermoscopy use, and in helping new users to develop their skills. Having another GP in the surgery who used dermoscopy was considered a very useful source of advice: *My colleague…is a good resource for me when I’m unsure of lesions’(I2)*.

For GPs without a colleague for advice, many turned to peer groups hosted on social media sites to post photographs and queries, for other members to respond with comments or advice. Generally participants were positive about these groups: *‘It’s always better to have second opinions…I’ve been surprised sometimes, you know, I’ve never even thought of that diagnosis at all…so, yeah, really helpful.’(I6)*.

However the limitations to advice from online groups were recognised, in particular that it was not equivalent to a formal opinion from another doctor: *‘You can’t ask people to put [a post onto an online group] on for an opinion because…there’s confidentiality, there’s insurance issues.’(I3)*.

#### Experience

GPs’ capability to use dermoscopy necessitated not only formal training, but also gaining practical clinical experience with dermoscopy. This was particularly important in building confidence so that GPs allowed their dermoscopy findings to influence their clinical decisions: *‘The more that I use a dermatoscope, the more likely I am to start to change my decisions, because I then develop an eye for, “I’m happy with that.”’(I8)*.

There was also evidence that previous experience in dermatology seemed to inspire these doctors with greater confidence in adopting new skills such as dermoscopy: *‘I think getting a good grounding [in dermatology] early on inspired me with confidence…you were confident to try things.’(I3)*.

#### Access

The cost of purchasing a dermatoscope was identified as a clear barrier to dermoscopy use. However access to a dermatoscope in the workplace seemed in itself to act as an incentive for non-users to upskill in it: *‘I’m now considering learning more about dermoscopy…simply by having the [dermatoscope].’(I2)*.

### Clinical impact

The second key factor influencing GPs’ use of dermoscopy was their impressions of its clinical impact. The impact of dermoscopy was discussed at the level of the GP consultation, the GP practice, and the wider health service.

#### Consultation

Many participants valued dermoscopy as a tool that provided a closer, more detailed examination of a lesion, and which seemed to help them reach a more accurate diagnosis: *‘Once you start to look down a dermatoscope…you certainly see more…and you just think, “Well, if I’m seeing more, I’m more likely to get the right diagnosis.”’(I8)* However there was a conflicting view among some participants who felt that the role of dermoscopy was limited as they already felt confident in the management of skin lesions: *‘Ninety-nine times out of a hundred you know to look at [a skin lesion] with your naked eye if it’s serious or not.’(I11)*.

The uncertainty that GPs deal with when managing skin lesions that are potentially skin cancers led some participants to use dermoscopy as a means of helping them to manage this risk: *‘[Dermoscopy] does give you a bit more reassurance…you can be more reassured if they’re benign, or if they are more sinister and they need to be referred on.’(I6)* However some participants, especially new dermoscopy users, felt unable to allow dermoscopy to influence their clinical decision-making: *‘I’m not confident enough with [dermoscopy] to really make a big decision.’(I2)*.

GPs generally felt that patients responded positively to their use of dermoscopy, that it felt like a more thorough examination, and therefore gave patients more confidence in the assessment: *‘I think [patients] are far more confident…that you have actually looked at [the skin lesion]…and [patients think], “Oh well he looked at it under the scope, so it’s alright.”’(I6)*.

Importantly, dermoscopy use did not seem to create extra work for GPs or lead to longer consultations: *‘It’s not adding to our workload in practice…but actually, to make our life easier here…I think it is quite useful.’(I7)*.

#### GP practice

Many participants felt that having a dermoscopy user in the GP practice provided a better service for their patients. Being able to refer patients to a colleague ‘in-house’ was appreciated by GPs, as it potentially avoided hospital referrals: *‘I think patients…would much prefer if someone in your own surgery was able to see [a skin lesion] than have to be referred on.’(I12)* Dermoscopy users did not feel that ‘in-house’ referrals added significantly to their workload: *‘I would have colleagues asking me to see a patient…it’s not a major impact on my day-to-day working, because it tends to be quick.’(I8)*.

However, there were concerns that using dermoscopy could unintentionally lead to the dermoscopy user being considered a ‘skin expert’ by colleagues or patients, when this was not the case: *‘[The GP dermoscopy user] might be the doctor that everybody would send their moles and skin lesions to, and some doctors might not necessarily want that.’(I5)* Additionally, there was concern that having another GP available to look a skin lesions might act as a disincentive for other GPs in the practice to start using dermoscopy: *‘Why do [other GPs] need to feel confident in [dermoscopy] whenever they can knock my door?…They don’t necessarily need to build up those skills.’(I8)*.

#### Health service

GPs recognised that specialist dermatology referral pathways were under pressure. Many GPs, especially established dermoscopy users, felt strongly that dermoscopy use reduced the number of dermatology referrals they made. Conversely, while newer dermoscopy users thought that dermoscopy made them more concerned about some skin lesions, even then, they reported that it helped them to triage how urgently referrals needed to be seen: *‘I don’t think it necessarily reduces the referrals, but…streamlines the referrals better, so, those ones which could be urgent and need to be seen, I think it’s better for highlighting that.’(I6)*.

There was general agreement that wider dermoscopy use among GPs could be cost-effective for the health service: *‘There’s no reason why the health and social care board wouldn’t provide funding for [dermoscopy training for GPs] because ultimately…it could save…on money being spent on outpatient clinics.’(I5)*.

### Acceptability

The third main factor influencing GPs’ use of dermoscopy was their impression of how appropriate dermoscopy is to a primary care setting. The predominant attitudes displayed by participants were of enthusiasm, reluctance, or scepticism.

#### Enthusiasm

Many participants expressed enthusiasm for the use of dermoscopy in primary care. It was acknowledged that GPs seem keen to upskill in this area, and that it complemented certain special interests such as minor surgery well: *‘I’m also a great believer in keeping your skills up to date and learning new skills…I don’t see any reason why GPs couldn’t learn [dermoscopy] and do it.’(I5)*.

#### Reluctance

Some participants, while not averse to the use of dermoscopy in primary care in principle, felt that it was not a priority, and therefore there was reluctance to take the necessary steps to introduce dermoscopy into practice. Reasons included having identified more urgent areas of clinical need within the practice, GPs being close to retirement, or considering dermoscopy complicated or time-consuming to learn: *‘It seemed quite an undertaking to train up in dermoscopy.’(I7)*.

#### Scepticism

Some participants expressed scepticism about whether the use of dermoscopy was well-suited to general practice. There was an impression by some that a dermatoscope was simply a magnifying glass. Others considered it preferable to refer to dermatologists, and there was also concern that dermoscopy training was being offered to GPs as an unwelcome alternative to improving referral pathways and access to specialist care: *‘I think the danger is that [training GPs in dermoscopy] is interpreted by GPs as:…what I want is a consultant dermatologist…but because that’s not available, I’ve been handed a dermatoscope and given a half-day’s training instead.’(I8)*.

## Discussion

### Summary

Three overarching factors influencing GPs’ use of dermoscopy were generated from semi-structured interview data by thematic analysis. GPs’ capability to use dermoscopy influenced use, and was determined by access to a dermatoscope, receiving adequate training, gaining practical experience with dermoscopy, and having some support for questions and queries. GPs’ perceptions of the clinical impact of dermoscopy on the GP consultation, on the wider GP practice and on the health service generally influenced its use. Finally perceptions of the acceptability of dermoscopy within primary care seemed to influence dermoscopy use, and these perceptions were heterogeneous, with enthusiasm, reluctance and scepticism all expressed by participants.

### Strengths and limitations

To our knowledge this is the first qualitative study assessing factors influencing GPs’ use of dermoscopy. Given the growing evidence for a potential role for dermoscopy in improving the assessment of suspected skin cancers [15, 16, 21], this work is therefore timely and important. This study sought to understand the factors influencing GPs’ use of dermoscopy to explore how dermoscopy use can be facilitated in primary care where appropriate. Its findings are therefore directly relevant to frontline primary care services.

The study was developed with reference to relevant qualitative research principles, and while the sampling strategy, in posting to an online group for GPs, may have excluded less technologically-savvy GPs, a varied sample was achieved. Furthermore, the range of views expressed by GPs, both dermoscopy users and non-users, including negative and sceptical views of dermoscopy, suggests that the study has captured a diversity of perceptions on the issues. Participants in this study were limited to GPs, but understanding patients’ perceptions of the role of dermoscopy in primary care would be important.

Generalising research findings must always be done with caution in qualitative research, and needs to be mindful of the structure of healthcare services, funding of primary care, GP training and geography of the study setting. Nevertheless it seems likely that pertinent findings in this study may be transferable to other comparable healthcare settings, especially given the resonance the results have with previous findings in the literature.

### Comparison with existing literature

The results of this study complement findings of previous studies, while adding important new insights. For example, while the cost of dermoscopy equipment and training has been previously recognised as a barrier to its use [25, 26], this study found evidence that having a dermatoscope available may in itself encourage dermoscopy non-users to begin using it. Furthermore, while there is evidence that dermoscopy improves GPs’ ability to triage pigmented skin lesions, and improves the accuracy of their skin lesion assessments [18, 19], some dermoscopy users in this study reported not feeling confident enough to allow dermoscopy to influence their clinical decisions, potentially limiting its usefulness.

Participants considered adequate training to be key in developing GPs’ capability in using dermoscopy, however some modalities of training are considered more useful than others [41]. In particular participants thought dermoscopy training needed to be hands-on, practical, and that short one-off training was inadequate. This concurs with the results of a scoping review looking at programs to train GPs in melanoma diagnosis, which found that the programs that led to sustained improvement in GPs’ clinical practice included refresher training material [42]; moreover the effectiveness of ongoing dermoscopy training for GPs delivered via distance learning has shown promising potential.[43] Conversely, a questionnaire study carried out among GPs with an interest in dermatology found that the majority reported feeling confident using dermoscopy after short-term training [27]. Our study may help to provide an explanation for this difference, in that it seems that previous experience in dermatology may give GPs more confidence in trying dermoscopy; GPs without prior dermatology experience may require more thorough dermoscopy training.

### Implications for research and practice

While GPs recognise the need for adequate training, the definition of this is unclear. Although well-established dermoscopy training courses exist, there are no agreed competency standards for dermoscopy use in the UK, either for GPs or for dermatologists [44, 45]. Developing competency standards could allow the impact of dermoscopy training to be assessed and allow different modalities of training to be compared for effectiveness.

Many participants, including dermoscopy non-users, considered dermoscopy to be an asset to a GP practice, and many suggested that dermoscopy be incorporated into GP training. Organisations such as PCDS recommends that ideally one GP in each practice would be able to use dermoscopy [32], but it is not presently part of the UK GP training curriculum [34], like it is in Australia where dermoscopy use among GPs is much more widespread [29, 33]. However any training programs must be sufficient to facilitate confident, effective, safe and long-term use of dermoscopy. The findings that experience in dermatology may facilitate the use of dermoscopy, and the perception among participants that dermoscopy skills need to be fostered within the context of more general dermatology training, stands in contrast to the very limited number to dermatology rotations available for GP trainees in the UK [1, 46].

This study has also highlighted several ethical issues that may arise with dermoscopy use in general practice. First is the impression that a GP is a ‘skin expert’: it is important that neither GPs nor their colleagues misrepresent their expertise, and regulators expect doctors to be honest about their experience and qualifications [47]. Indeed being unwittingly considered an expert was considered a potential barrier to dermoscopy use among participants in this study. Second is the use of online peer groups to post queries, which many GPs use as a form of professional and educational support. However any reliance on these groups on the part of GPs to aid clinical decision-making would raise concerns about patient safety and quality of care, given that the expertise of those posting and responding to queries online is unknown. Furthermore many of these groups are multinational in composition, and standards of practice may vary between countries, for example, on whether the excision of suspected melanomas in primary care is accepted practice. Moreover the capturing and posting of patient images for training or teaching purposes raises important issues of consent, confidentiality and data protection. GPs must be aware of relevant guidance on these issues and consider any unintended risks to patients [48, 49].

## Conclusions

This qualitative study has revealed several important factors influencing GPs’ use of dermoscopy. While the limitations of a qualitative methodology must be acknowledged when interpreting and generalising study findings, this study design was important in gaining new insights that have not been previously identified in the published literature. One such finding was the need for adequate training in dermoscopy, which was emphasised as a key barrier to its use, and highlighted that existing training, including one-off training days, is often considered insufficient by GPs. The absence of agreed competency standards for the use of dermoscopy in general practice makes the impact of training programs difficult to assess and compare at present.

Both GPs who use dermoscopy, and those who do not, consider it to have an important role in improving skin assessments within primary care, in agreement with previous studies which have shown that dermoscopy use may help GPs to assess and triage suspect skin lesions more accurately. However translating these positive perceptions of dermoscopy into greater use of dermoscopy among GPs will require additional barriers such as high equipment costs and low confidence in dermatology to be addressed. The use of online peer groups to post queries and photographs raises several important issues around good medical practice that need to be addressed so that their use can be both beneficial for clinicians and safe for patients.

Given the significant proportion of skin assessments undertaken by GPs, these findings are relevant both to those working in primary care, and those providing funding and training to GPs and GP trainees.

## Data Availability

The datasets generated and analysed during the current study are not publicly available to avoid any potential for participants to be identified, but are available from the corresponding author on reasonable request.

## Abbreviations

GP: General Practitioner
NHS: National Health Service (UK)
NI: Northern Ireland
PCDS: Primary Care Dermatology Society
